# *Lactobacillus delbrueckii* Ameliorates Intestinal Integrity and Antioxidant Ability in Weaned Piglets after a Lipopolysaccharide Challenge

**DOI:** 10.1155/2020/6028606

**Published:** 2020-02-10

**Authors:** Fengming Chen, Houjun Wang, Jiayi Chen, Yang Liu, Wei Wen, Yinghui Li, Xingguo Huang

**Affiliations:** ^1^College of Animal Science and Technology, Hunan Agricultural University, Changsha Hunan 410128, China; ^2^Hunan Co-Innovation Center of Animal Production Safety (CICAPS), Changsha Hunan 410128, China

## Abstract

This study was conducted to evaluate the effect of dietary supplementation with *Lactobacillus delbrueckii* (LAB) on intestinal morphology, barrier function, immune response, and antioxidant capacity in weaned piglets challenged with lipopolysaccharide (LPS). A total of 36 two-line crossbred (Landrace × large Yorkshire) weaned piglets (28 days old) were divided into three groups: (1) nonchallenged control (CON); (2) LPS-challenged control (LPS); and (3) LAB+LPS treatment (0.2% LAB+LPS). Compared to the LPS piglets, the LAB+LPS piglets improved intestinal morphology, indicated by greater (*P* < 0.05) villus height in the duodenum and ileum; villus height : crypt depth ratio in the duodenum, jejunum, and ileum, as well as decreased (*P* < 0.05) crypt depth in the jejunum and ileum; and better intestinal barrier function, indicated by upregulated (*P* < 0.05) mRNA expression of tight junction proteins in the intestinal mucosa. Moreover, compared to the LPS piglets, LAB significantly decreased (*P* < 0.05) concentrations of TNF-*α* and IL-1*β* in the small intestine and increased (*P* < 0.05) IL-10 levels in the jejunum and ileum. Additionally, LAB increased (*P* < 0.05) T-AOC activities of the colon, GSH concentrations of the jejunum, and mRNA expression of CAT and Cu/Zn-SOD, while reduced (*P* < 0.05) MDA concentrations in the jejunum and ileum in LPS-changed piglets. Collectively, our results indicate that supplementation of LAB improved intestinal integrity and immune response and alleviated intestinal oxidative damage in LPS-challenged piglets.

## 1. Introduction

The maintenance of cellular redox homeostasis is imperative for the survival and normal function of cells. However, the excessive production of reactive oxygen species (ROS) or poor capability of scavenging reactive intermediates is considered causing a continuous imbalance in redox homeostasis, which resulted in endogenous oxidative stress [1, 2]. Many studies have shown that oxidative stress causes DNA hydroxylation, protein denaturation, lipid peroxidation, and intestinal injury [3]. Considerable research revealed that *Escherichia coli* Lipopolysaccharide (LPS) could induce the formation of ROS intermediates, which contributes to a part of LPS toxicity [4]. Therefore, LPS has long been used to induce oxidative stress in experimental animals [5].

In commercial swine production, weaning stress is especially severe during the first week after weaning, which often results in “postweaning stress syndrome” [6]. An increasing number of studies have found weaning could disrupt the physiologic equilibrium of oxidant, which may lead to oxidative stress and eventually induce enterocyte apoptosis and cell cycle arrest in the small intestine of postweaning piglets [7, 8]. Oxidative stress has been proved to be involved in intestinal barrier dysfunction and various digestive tract diseases [9]. The adverse effects of oxidative stress on intestinal morphology are accompanied by downregulation of expression in tight junction protein [10, 11]. Through the study of an epithelial cell model in vitro, it was found that oxidative stress could reduce occludin and ZO-1 mRNA expression and destroy their distribution [12]. Besides, oxidative stress is thought to play a key role in the development of intestinal damage in inflammatory bowel disease. Activated leukocytes not only generate a wide range of proinflammatory cytokines but also participate in excessive oxidative reactions, which significantly change the redox balance in the intestinal mucosa and maintain inflammation by inducing redox-sensitive signaling pathways and transcription factors. Furthermore, several inflammatory molecules generate ROS, leading to a self-sustaining and autoamplifying vicious circle, which eventually impairs the structure and function of the intestinal barrier [13].

It is widely acknowledged that probiotic microbes, especially *Lactobacillus* species, are essential in regulating the homeostasis of intestinal microecology, as well as improving the function of mucosal barrier and alleviating the symptoms of intestinal diseases [14, 15]. Our team has proved the benefit of *Lactobacillus delbrueckii* (LAB, a kind of lactic acid bacteria) on improving the antioxidant status and stimulating the immune response in suckling piglets at the amounts of 1, 2, 3, and 4 mL at 1, 3, 7, and 14 d of age [16]. Based on this finding, we hypothesized that LAB could ameliorate *Escherichia coli* LPS-induced intestinal integrity, immune function, and antioxidant capacity in weaned piglets. In the present study, we established an oxidative stress model by injecting LPS, in order to investigate whether LAB administration could improve LPS-induced intestinal injury and health condition via regulating intestinal morphology, barrier function, immune response, and antioxidant capacity.

## 2. Materials and Methods

### 2.1. Bacterial Strain

The strain LAB CCTCC M 207040 was obtained from the microbiology laboratory of the College of Animal Science and Technology, Hunan Agricultural University. The LAB were incubated in a stationary state at 37°C for 48 h in de Man, Rogosa and Sharp (MRS) medium in an anaerobic condition. The viable counts in culture medium were determined by the gradient dilution coating method, stored at 4°C, measured, and adjusted to 50 × 10^8^ colony-forming units (CFU)/mL. After incubation and counting of colony-forming units, 1.0 L cultured medium was mixed with 0.25 kg wheat bran and then freeze-dried [17]. In the present study, the potent preparation of LAB was added to the experimental diet at a dose of 2.01 × 10^10^ CFU/g.

### 2.2. Animals, Diet, and Housing

The experimental protocols and procedures performed in this study were approved by the Animal Welfare Committee of Hunan Agricultural University. A total of 36 two-line crossbred (Landrace × large Yorkshire) piglets weaned at 28 d of age were randomly allocated into 3 treatments with 6 replicate pens of 2 piglets. The corn-soybean basal diet (Table 1) was formulated in powder without any in-feed antibiotics according to NRC (2012) requirements for all nutrients [18]. Piglets were housed in a temperature-controlled nursery and had ad libitum access to feed and water throughout the 28 d feeding trial.

### 2.3. Experimental Procedures

Treatments included the following: (1) nonchallenged control (CON; piglets were fed a basal diet and injected with sterile saline); (2) LPS-challenged control (LPS; piglets were fed the same basal diet and challenged by injection with Escherichia coli LPS); and (3) LAB+LPS treatment (0.2% LAB+LPS; piglets were fed the basal diet supplemented with 0.2% of LAB and challenged by LPS). LAB (powder) was mixed with the premix feed and then transferred into a larger mixer (total capacity 500 kg) where the final volume of the weekly feed batch was prepared. At 8:00 on day 29, the challenged group was intraperitoneally administered with LPS (*Escherichia coli* O55:B5, Sigma Chemical) at 100 mg/kg body weight (BW) and the unchallenged group received an equivalent amount of 0.9% (*w*/*v*) NaCl solution. The dose of LPS was chosen based on previous studies with weaned piglets [19]. Piglets were weighed individually on day 29 followed by challenging immediately. Troughs were weighed, and feed was added daily to determine pen feed intake. The average daily gain (ADG), average daily feed intake (ADFI), and the ratio of feed and gain (F : G) were calculated per pen.

### 2.4. Sample Collection

Six piglets (1 per replicate) randomly selected from each treatment were humanely killed by intravenous injection of sodium pentobarbital (40 mg/kg body weight) 3 h after injection of LPS or saline. Blood samples were collected in 10 mL tubes and centrifuged at 3000 g for 15 min at 4°C, and the supernatants (serum) were collected and stored at -80°C until subsequent analysis. The gastrointestinal tracts were collected on a chilled stainless steel tray, and the small intestine was separated from the large intestine and the mesentery. The 10 cm segments were cut at the distal duodenum, midjejunum, midileum and midcolon, respectively. The contents were flushed with ice-cold phosphate-buffered saline and divided into two sections. On one section (approximately 2 cm) segment, the duodenum, jejunum, and ileum were collected in 4% paraformaldehyde in phosphate-buffered saline (PBS) for subsequent histologic analysis; the other one was used for collecting the mucosa. The mucosal cell layers were gently scraped with a glass slide and then quickly placed in liquid nitrogen and stored at -80°C until analysis.

### 2.5. Intestinal Morphology Analysis

Villus height and crypt depth were measured on hematoxylin and eosin (H&E) stain and paraformaldehyde-fixed ileal histological slices. A detailed method of these tests has been described previously [20].

### 2.6. Measurement of Intestinal Mucosal Cytokines

Intestinal mucosa cytokines, including tumor necrosis factor-alpha (TNF-*α*), interleukin 1 beta (IL-1*β*), and interleukin 10 (IL-10), were determined using ELISA Kits according to the manufacturer's instructions (Cusabio Biotech Co., Ltd., Hubei, China), using the methods as we have described in detail previously [16].

### 2.7. Determination of Antioxidant Indices

Superoxide dismutase (SOD), glutathione peroxidase (GSH-Px), total antioxidant capability (T-AOC), malondialdehyde (MDA), glutathione (GSH), and hydrogen peroxide (H_2_O_2_) of the serum and intestinal mucosa were measured using commercial assay kits (Nanjing Jiancheng Bioengineering Institute, Nanjing, China) according to the manufacturer's recommendations. Protein concentration in the intestinal mucosa was determined following the manufacturer's instructions (bicinchoninic acid assay; Beyotime Biotechnology, Beijing, China). Intestinal mucosal antioxidant indices were standardized to the protein in each sample.

### 2.8. Relative Quantification of mRNA Expression of Antioxidative Enzymes and Tight Junction Protein

RNA was extracted from each tissue sample (100 mg) using the TRIzol Reagent (Invitrogen Life Technologies, USA) according to manufacturer's guidelines. RNA concentration was measured using a Nano Drop spectrophotometer (ND-2000; NanoDrop Technologies, Wilmington, DE, USA). Total RNA (1 *μ*g) from each sample was reverse transcribed into cDNA using PrimeScript™ RT reagent Kit with a TC-512 PCR system (TECHNE, UK). The levels of mRNA expression were quantified by real-time PCR with SYBR™ Premix Ex Taq™ II (TaKaRa, Japan) and ABI7900HT Real-Time PCR System (Applied Biosystems, USA). The primers were designed with the use of Primer 5.0 according to the gene sequence of pigs to produce amplification products (Table 2). Relative gene expression was expressed as a ratio of the target gene to the control genes using the following formula: 2^‐(ΔΔCt)^, where *ΔΔ*Ct = (Ct_target_ ‐ Ct_*β*-actin_)_treatment_ ‐ (Ct_target_ ‐ Ct_*β*-actin_)_control_. All samples were run in triplicate.

### 2.9. Statistical Analysis

The results were presented as the means ± standard error of the mean (SEM). Pens were considered the experimental unit for growth performance, whereas individual pig was used as the experimental unit for the analysis of intestinal and serumal data. The statistical significance was analyzed using one-way analysis of variance (ANOVA), followed by Duncan's multiple range test using SPSS 19.0 (Chicago, IL, USA). Probability values ≤ 0.05 were taken to indicate significance.

## 3. Results

### 3.1. Growth Performance

Throughout the 28 d feeding trial (prechallenge), there were no differences in initial (7.79 ± 0.96 kg) and final BW (16.15 ± 1.16 kg), daily gain (299.20 ± 30.91 g), daily feed intake (430.93 ± 23.60 g), or the gain : feed ratio (0.69 ± 0.03) among the three groups.

### 3.2. Intestinal Morphology

Data on the intestinal morphology are summarized in Table 3. Compared to the CON, piglets challenged with LPS exhibited a decrease (*P* < 0.05) in villus height and the ratio of villus height to crypt depth at all the sites, as well as an increase (*P* < 0.05) in crypt depth in the jejunum and ileum. Compared to the LPS piglets, dietary supplementation of 0.2% LAB significantly increased (*P* < 0.05) villus height in the duodenum (12.63% higher) and ileum (15.83% higher) and the ratio of villus height to crypt depth in the duodenum (20.93% higher), jejunum (31.68% higher), and ileum (72.41% higher), while it decreased crypt depth in the jejunum (22.32% lower) and ileum (31.76% lower).

### 3.3. mRNA Expression of Tight Junction Protein in Intestinal Mucosal

**Figure 1:**
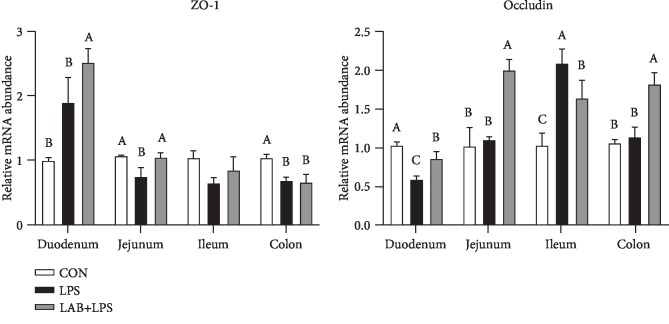
Effects of dietary *Lactobacillus delbrueckii* supplementation on tight junction protein mRNA expression in the intestinal mucosa of piglets after LPS challenge. Data are the mean ± SEM (*n* = 6). CON (nonchallenged control)—piglets were fed the basal diet and injected with sterile saline; LPS (LPS-challenged control)—piglets were fed the basal diet and challenged with *Escherichia coli* LPS; LAB+LPS (0.2% LAB+LPS)—piglets were fed the basal diet supplemented with 0.2% LAB and challenged with LPS. ^A,B,C^Within the same intestinal segment, means with different superscript letters differ (*P* < 0.05).

Figure 1 shows that LPS challenge downregulates (*P* < 0.05) the expression levels of ZO-1 in the jejunum and colon compared with the CON group. In comparison with the LPS piglets, dietary supplementation with 0.2% LAB increased (*P* < 0.05) ZO-1 mRNA expression in the duodenum and jejunum. In contrast with the CON group, intraperitoneal administration of LPS decreased (*P* < 0.05) the mRNA expression levels of occludin in the duodenum but did not affect (*P* > 0.05) occludin expression in the jejunum and colon. Additionally, the mRNA expressions of occludin in the jejunum and colon of the LAB+LPS group were higher (*P* < 0.05) than those of the CON and LPS groups.

### 3.4. Intestinal Mucosal Cytokines

**Figure 2:**
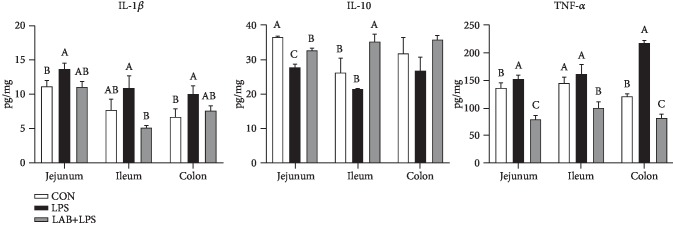
Effects of dietary *Lactobacillus delbrueckii* supplementation on concentrations of cytokines (IL-1*β*, IL-10 and TNF-*α*) in the intestinal mucosal of piglets after LPS challenge. Data are the mean ± SEM (*n* = 6). CON (nonchallenged control)—piglets were fed the basal diet and injected with sterile saline; LPS (LPS-challenged control)—piglets were fed the basal diet and challenged with *Escherichia coli* LPS; LAB+LPS (0.2% LAB+LPS)—piglets were fed the basal diet supplemented with 0.2% LAB and challenged with LPS. ^A,B,C^Within the same intestinal segment, means with different superscript letters differ (*P* < 0.05).

The results obtained of the concentrations of cytokines in the intestinal mucosa are presented in Figure 2. Compared to the CON group, the LPS piglets exhibited an increase (*P* < 0.05) in the concentrations of TNF-*α* and IL-1*β* in both the jejunum and colon. Relative to the LPS group, the piglets fed with 0.2% LAB had lower (*P* < 0.05) TNF-*α* concentrations in the jejunum, ileum, and colon. Piglets challenged with LPS had lower (*P* < 0.05) concentrations of IL-10 in the jejunum than those in the CON group. However, the piglets fed the LAB diet had higher (*P* < 0.05) IL-10 concentrations in the jejunum and ileum compared with the LPS piglets.

### 3.5. Antioxidant Activities of the Serum and Intestinal Mucosa

**Figure 3:**
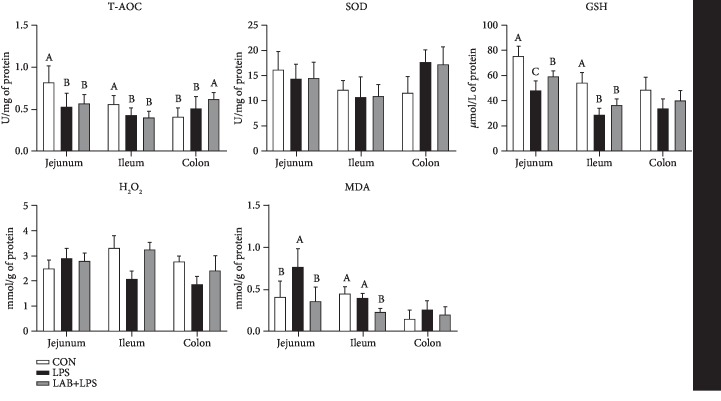
Effects of dietary *Lactobacillus delbrueckii* supplementation on antioxidant activities in the intestinal mucosa of piglets after LPS challenge. Data are the mean ± SEM (*n* = 6). CON (nonchallenged control)—piglets were fed the basal diet and injected with sterile saline; LPS (LPS-challenged control)—piglets were fed the basal diet and challenged with *Escherichia coli* LPS; LAB+LPS (0.2% LAB+LPS)—piglets were fed the basal diet supplemented with 0.2% LAB and challenged with LPS. ^A,B,C^Within the same intestinal segment, means with different superscript letters differ (*P* < 0.05).

Antioxidant activities of the serum and intestinal mucosa are shown in Table 4 and Figure 3, respectively. Compared to the CON, the T-AOC activity in the serum, jejunum, and ileum; GSH-Px activities in serum; and GSH levels in the jejunum and ileum were decreased (*P* < 0.05) in piglets after LPS challenge. The MDA and H_2_O_2_ concentrations in the serum and jejunum were increased (*P* < 0.05) in piglets after LPS challenge as well. In comparison with the LPS piglets, dietary supplementation of LAB increased (*P* < 0.05) the T-AOC activity in the serum and colon, GSH-Px activity in serum, and GSH concentrations in the jejunum, while it decreased (*P* < 0.05) the MDA and H_2_O_2_ concentrations in serum and MDA concentrations in the jejunum and ileum. However, there were no significant differences in the SOD activities in the serum and mucosa among the three groups.

### 3.6. mRNA Expression of Anti-Oxidative Enzymes in Intestinal Mucosa

**Figure 4:**
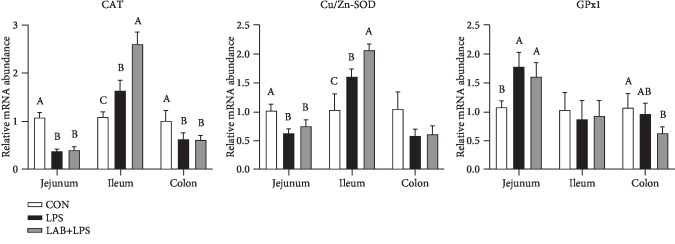
Effects of dietary *Lactobacillus delbrueckii* supplementation on antioxidative enzymes mRNA expression in the intestinal mucosa of piglets after LPS challenge. Data are the mean ± SEM (*n* = 6). CON (nonchallenged control)—piglets were fed the basal diet and injected with sterile saline; LPS (LPS-challenged control)—piglets were fed the basal diet and challenged with *Escherichia coli* LPS; LAB+LPS (0.2% LAB+LPS)—piglets were fed the basal diet supplemented with 0.2% LAB and challenged with LPS. ^A,B,C^Within the same intestinal segment, means with different superscript letters differ (*P* < 0.05).

To further reveal the molecular mechanism of dietary probiotic LAB implicated in regulating antioxidant capacity in the intestinal mucosa, mRNA expressions of antioxidative enzymes (CAT, Cu/Zn-SOD, and GPx1) were determined, as shown in Figure 4. Challenge with LPS caused a reduction (*P* < 0.05) in mRNA expression of CAT in both the jejunum and colon as well as Cu/Zn-SOD in the jejunum, and LPS tended to decrease (*P* > 0.05) the colonic Cu/Zn-SOD mRNA expression compared with the CON group. On the contrary, supplementation with LAB resulted in a significant increase in CAT and Cu/Zn-SOD mRNA expression in the ileum.

## 4. Discussion

Probiotics such as *Lactobacillus* have been reported to improve the intestinal microecological environment, stabilize intestinal mucosal barrier, prevent ROS-mediated oxidative stress of intestine, and alleviate the symptoms of intestinal diseases [21, 22]. Accordingly, we investigated the protective effect of 0.2% of LAB on intestinal integrity and antioxidant ability after a 3 h Escherichia coli LPS challenge using a piglet model. Results of the present study illustrated that dietary supplementation with LAB had no effects on ADFI, ADG, and G : F ratio of weaned piglets. Similar observations were obtained by Strompfova et al. [23] and Herfel et al. [24], indicating that there were no effects of the selected probiotic strains on the growth performance of piglets. However, there have been several other attempts to demonstrate the use of probiotics as potential alternatives to antimicrobial growth promoters. For instance, dietary probiotic supplementation was shown to improve growth performance in pigs (dose of *Bacillus organisms* was 1.47 × 10^8^ CFU/g) [25] and broilers (dose of *Saccharomyces boulardii and Bacillus subtilis B10* was 1 × 10^8^ CFU/kg) [26], due to the fact that probiotics improved the digestibility of nutrients by increasing the production and activities of digestive enzymes [25]. The discrepancies may be caused by the differences in the species and ages of animals, as well as the strains and dose of probiotics. Also, differences in the mode of probiotic application and time of application may be contributing factors.

Intestinal morphology is one of the essential indicators to reflect the health status of the gut [27]. Villus height and crypt depth are associated with intestinal morphology, reflecting the number and maturation rate of enterocytes. Morphological changes in the digestive tract and permeability increase in the mucosal caused by LPS have been widely acknowledged [28, 29]. On the other hand, the intestinal epithelial barrier is primarily composed of epithelial cells and the tight junctions between them [30]. The tight junctions are composed of transmembrane proteins (claudins, occludin, and E-cadherin), cytoplasmic protein (ZO-1), and many other regulatory proteins [31], which regulate lateral intercellular permeability and contribute to the integrity of epithelial barrier function [32]. As expected, the LPS challenge resulted in a decrease of small intestinal villus height and an increase of the crypt depths in jejunum and ileum in our study, which suggests that LPS caused intestinal injury in piglets. These adverse effects on intestinal morphology by LPS were attenuated by LAB supplementation, which is in agreement with an earlier finding that dietary 2 × 10^9^ CFU/mL *Lactobacillus fermentum* I5007 supplementation significantly increased jejunal villus height in piglets on day 14 [33]. We further confirmed that LAB significantly attenuated the decrease in ZO-1 expression in the jejunum and the increase in occludin expression in the jejunum and colon in piglets caused by LPS challenge, which demonstrates that dietary LAB helped to improve the intestinal integrity and barrier function. The results were directly compared with the previous study [34] showing that dietary 2 × 10^9^ CFU/mL *L. reuteri* I5007 supplementation effectively improved the intestinal mucosal barrier function of newborn piglets via upregulating the expression of occludin and ZO-1 in the jejunal epithelial and the expression of claudin-1, occludin, and ZO-1 in the ileal epithelial. In fact, one of the effects probiotics exert is to promote the integrity of the intestinal mucosal barrier by affecting the expression and structure of tight junction proteins [34].

Oxidative stress is closely related to chronic inflammation [35]. It is well known that cytokines produced in the body's immune process mainly included proinflammatory cytokines (IL-1*β* and TNF-*α*) and anti-inflammatory molecules (IL-10). In this study, LPS-challenged piglets exhibited an increase in TNF-*α* and IL-1*β* concentrations in all three intestinal segments and a decrease in IL-10 concentration in the jejunum and ileum. This result indicates a serious inflammation response in the intestine induced by LPS, which is in general agreement with the increased expression of proinflammatory cytokines in the intestine of LPS-challenged humans and animals [29, 36]. It is reported that probiotics played an immunomodulatory role by releasing various cytokines from immune cells [37]. Karamese et al. [38] found that a mixture of *Lactobacillus* and *Bifidobacterium* species played a vital role in regulating immune responses in rats through the upregulation of IL-10 and the downregulation TNF-*α* and IL-6. Another experiment demonstrated that *Lactobacillus casei* and *Lactobacillus bulgaricus* significantly reduced the TNF-*α* production in the mucosa of Crohn's disease patients [39]. What is more, previous studies indicated that overproduction of proinflammatory cytokines could cause a negative influence on intestinal mucosal integrity, permeability, and epithelial functions [40]. Compared to the LPS piglets, the production of TNF-*α* and IL-1*β* was decreased in the intestinal mucosa, while IL-10 concentrations were increased in the jejunum and ileum of LAB-treated piglets in this study, confirming the roles of probiotic LAB in the intestinal integrity and epithelial function. Similar to our results, many studies have reported that probiotic administration suppressed intestinal inflammation by the reduction in TNF-*α* and IL-1*β* concentrations in the intestine [41, 42]. This result indicated a protective effect of the LAB against LPS-induced intestinal inflammation and supported the hypothesis that the LAB has beneficial effects in reducing intestinal mucosal inflammation.

It is quite evident that oxidative stress is capable of destroying the host antioxidant system and the cellular redox homeostasis [43]. Normally, ROS can be scavenged by an antioxidant defense system mainly including antioxidant enzymes, such as SOD and GSH-Px, and other nonenzymatic antioxidants (typically glutathione) [44]. H_2_O_2_, a major type of ROS and a molecular culprit inducing lipoperoxidation, is synthesized via a dismutation reaction catalyzed by SOD and transformed into water by GSH-Px or CAT [45]. As the end product of lipid peroxidation, MDA is often regarded as the index for oxidative injury [46]. A large body of evidence has demonstrated that LPS increases the production of reactive oxygen intermediates such as superoxide radical, lipid peroxides, and nitric oxide, eventually causing oxidative damage [4]. In the current study, T-AOC activity in the serum, jejunum, and ileum; GSH-Px activities in the serum; and GSH levels in the jejunum and ileum were decreased, and MDA and H_2_O_2_ concentrations in the serum and jejunum were increased in piglets after LPS challenge. These indicated a successful establishment of the oxidative stress model with the LPS challenge. Accumulated evidences have shown that *Lactobacillus* strains can improve antioxidant activities and biological functions in different animals [16, 47]. Research in pigs showed that dietary 1.02 × 10^8^ CFU/g of *Lactobacillus fermentum* supplementation could increase SOD and GPx activity of serum, muscle SOD activity, and Cu/Zn-SOD activity [48]. Shen et al. [49] also reported that *Lactobacillus plantarum* enhanced antioxidant capacities in the serum and liver of broilers by increasing GSH-Px activities and decreasing MDA concentrations. Similarly, the present study confirmed these findings as our data showed that dietary LAB supplementation could increase T-AOC activity in the serum and colon, GSH-Px activity in the serum, and GSH concentration in the jejunum, as well as decreased MDA and H_2_O_2_ concentrations in serum and MDA concentrations in the jejunum and ileum. Moreover, we further confirmed that a significant increase in the mRNA expression of antioxidant enzymes, including CAT and Cu/Zn-SOD in the ileal mucosa of LAB+LPS piglets, displaying less oxidative stress of piglets, indicates the antioxidant ability of LAB and contributes to improved intestinal health. However, the physiological mechanism of the antioxidant capacity of probiotics has not been correctly revealed [50], and whether LAB has a direct or indirect effect on enzyme activity is not clear [16].

In summary, our findings demonstrated that probiotic LAB is effective to improve intestinal morphology, barrier function, immune response, and antioxidant ability of piglets challenged with LPS. Consequently, the LAB might play an important role in alleviating intestinal oxidative damage of piglets induced by weaning stress. These findings not only aid in understanding the LAB's mode of actions in the gut of piglets but also have broad applications in relieving oxidative stress-related diseases in animal husbandry. However, further studies are still required to elucidate the molecular mechanisms underlying the protective role of LAB.

## Figures and Tables

**Table 1:** Ingredient composition of the basal diet (as-fed basis).

Item	Content
Ingredient (%)	
Extruded corn	50.00
Soybean meal (44% crude protein)	19.20
Extruded soybean	13.52
Fish meal	3.00
Whey powder	10.00
L-Lysine HCl (78%)	0.35
L-Methionine (98%)	0.21
L-Threonine (98%)	0.11
Dicalcium phosphate	0.91
Limestone	0.70
Vitamin and mineral premix^a^	2.00
Nutrient composition^b^	
Digestible energy (MJ/kg)	14.58
Crude protein (%)	19.21
Calcium (%)	0.78
Available phosphorus (%)	0.45
Lysine (%)	1.38
Methionine (%)	0.52
Threonine (%)	0.86

^a^Premix supplied per kg diet: retinyl acetate, 5512 IU; cholecalciferol, 2200 IU; DL-*α*-tocopheryl acetate, 30 IU; menadione sodium bisulfite complex, 4 mg; riboflavin, 5.22 mg; D-calcium-pantothenate, 20 mg; niacin, 26 mg; vitamin B 12, 0.01 mg; Mn (MnSO_4_·H_2_O), 63.6 mg; Fe (FeSO_4_·H_2_O), 90 mg; Zn (ZnSO_4_·7H_2_O), 75 mg; Cu (CuSO_4_·5H_2_O), 100 mg; I (CaI_2_), 0.2 mg; Se (Na_2_SeO_3_), 0.2 mg. ^b^Calculated value.

**Table 2:** Primers used for real-time PCR analysis.

Accession no.	Gene	Primers	Product length (bp)
DQ845171.1	*β*-Actin	F: CTGCGGCATCCACGAAACT R: AGGGCCGTGATCTCCTTCTG	147
XM_003353439.2	ZO-1	F: CCTGCTTCTCCAAAAACTCTT R: TTCTATGGAGCTCAACACCC	252
NM_001163647.2	Occludin	F: ACGAGCTGGAGGAAGACTGGATC R: CCCTTAACTTGCTTCAGTCTATTG	238
NM_214201	GPx1	F: TGGGGAGATCCTGAATTG R: GATAAACTTGGGGTCGGT	183
NM_001190422	Cu/Zn-SOD	F: CAGGTCCTCACTTCAATCC R: GATAAACTTGGGGTCGGT	255
NM_214301.2	CAT	F: CGAAGGCGAAGGTGTTTG R: AGTGTGCGATCCATATCC	374

**Table 3:** Effects of dietary *Lactobacillus delbrueckii* supplementation on the intestinal morphology of piglets after LPS challenge.

Items	CON	LPS	LAB+LPS
Villus height (*μ*m)			
Duodenum	376.37 ± 27.44^a^	255.82 ± 20.07^c^	288.12 ± 24.37^b^
Jejunum	332.25 ± 27.39^a^	252.73 ± 8.62^b^	260.73 ± 28.37^b^
Ileum	248.37 ± 24.06^a^	188.75 ± 13.39^c^	218.62 ± 10.71^b^
Crypt depth (*μ*m)			
Duodenum	107.63 ± 13.30	103.32 ± 15.04	91.93 ± 8.02
Jejunum	84.75 ± 10.12^b^	126.37 ± 14.27^a^	98.17 ± 11.35^b^
Ileum	77.13 ± 16.48^b^	130.30 ± 6.65^a^	88.92 ± 10.83^b^
Villus height/crypt depth			
Duodenum	3.41 ± 0.45^a^	2.58 ± 0.37^b^	3.12 ± 0.27^a^
Jejunum	3.99 ± 0.56^a^	2.02 ± 0.25^c^	2.66 ± 0.13^b^
Ileum	3.35 ± 0.87^a^	1.45 ± 0.13^c^	2.50 ± 0.35^b^

Data are the mean ± SEM (*n* = 6). CON (nonchallenged control)—piglets were fed the basal diet and injected with sterile saline; LPS (LPS-challenged control)—piglets were fed the basal diet and challenged with *Escherichia coli* LPS; LAB+LPS (0.2% LAB+LPS)—piglets were fed the basal diet supplemented with 0.2% LAB and challenged with LPS. ^a,b,c^Values within a row with different letters differ (*P* < 0.05).

**Table 4:** Effects of dietary *Lactobacillus delbrueckii* supplementation on serum antioxidant indices of piglets after LPS challenge.

Items	CON	LPS	LAB+LPS
T-AOC (U/mL)	5.34 ± 0.80^ab^	4.90 ± 0.76^b^	6.43 ± 0.96^a^
SOD (U/mL)	4.73 ± 0.58	4.89 ± 0.60	5.03 ± 0.54
GSH-Px (U/mL)	604.49 ± 30.58^a^	494.17 ± 40.60^b^	596.89 ± 61.24^a^
GSH (*μ*mol/L)	7.91 ± 3.13	7.14 ± 2.42	7.70 ± 2.38
MDA (mmol/L)	2.18 ± 0.19^c^	2.60 ± 0.18^a^	2.28 ± 0.19^b^
H_2_O_2_ (mmol/L)	40.98 ± 6.29^b^	55.31 ± 10.26^a^	28.70 ± 5.30^c^

Data are the mean ± SEM (*n* = 6). CON (nonchallenged control)—piglets were fed the basal diet and injected with sterile saline; LPS (LPS-challenged control)—piglets were fed the basal diet and challenged with *Escherichia coli* LPS; LAB+LPS (0.2% LAB+LPS)—piglets were fed the basal diet supplemented with 0.2% LAB and challenged with LPS. ^a,b,c^Values within a row with different letters differ (*P* < 0.05).

## Data Availability

The data used to support the findings of this study are available from the corresponding author upon request.
